# White Coat Hypertension in Primary Care: A Narrative Review

**DOI:** 10.7759/cureus.95256

**Published:** 2025-10-23

**Authors:** Arnaz Avasia, Madhavi Medipally, Mruganka Parasnis

**Affiliations:** 1 Public Health, University of Alabama at Birmingham, Birmingham, USA; 2 Family Medicine, University of Massachusetts, Amherst, USA; 3 Biomedical Engineering, Tufts University, Medford, USA

**Keywords:** ambulatory blood pressure monitoring, office blood pressure, out-of-office blood pressure, patient anxiety, patient-physician relationship, white coat effect, white coat hypertension

## Abstract

White coat hypertension (WCH) is the presence of elevated blood pressure (BP) in clinical settings, despite normal readings outside of the office, measured through ambulatory BP monitoring (ABPM) or home BP monitoring (HBPM), in untreated individuals. WCH is associated with increased risk of sustained hypertension (SH), target organ damage (TOD), metabolic syndrome, and cardiovascular (CV) events. The prevalence of WCH is not consistent across studies due to different BP cut-offs used and varies depending on the definition of WCH and the cohort studied. There is no universally defined approach to its definition, with variability among the American College of Cardiology (ACC)/American Heart Association (AHA), European Society of Hypertension (ESH), and National Institute for Health and Care Excellence (NICE) guidelines.

There is insufficient evidence from randomized controlled trials on whether WCH requires treatment. Current guidelines suggest lifestyle interventions and monitoring for patients with no additional CV risk factors, and lifestyle interventions along with drug treatment for those patients with increased CV risk or with hypertension-mediated organ damage. Annual ABPM or HBPM is advised to track progression to SH. Studies have shown that WCH may not be benign, with some evidence linking it to an increased risk of CV events and total mortality.

Several barriers hinder effective diagnosis in primary care, including cost, limited access to ABPM, provider knowledge gaps, and patient non-compliance with HBPM protocols. Improvement strategies include health provider training, patient education, enhanced physician-patient communication, and potential use of telemedicine for remote BP monitoring. Strengthening the patient-physician relationship may also reduce anxiety-driven BP elevations during clinic visits.

Addressing these barriers through policy changes and behavioral interventions can enhance the identification and management of WCH, prevent overtreatment, and reduce healthcare costs.

## Introduction and background

White coat hypertension (WCH) is a blood pressure (BP) phenotype present in individuals, characterized by increased BP readings in the clinic setting in the presence of a health care provider and normal out-of-office BP levels [1,2]. The European Society of Hypertension (ESH)/Society of Cardiology guidelines suggest that WCH is characterized by office BP readings of 140/90 mmHg or higher on at least three occasions, with a normal 24-hour ambulatory BP measurement of <130/80 mmHg or a home BP reading of 135/85 mmHg. However, due to failure to diagnose WCH appropriately with standardized measurements, there is overuse of antihypertensive medications in individuals who do not have persistent hypertension [1,2]. There is inadequate evidence from randomized controlled trials on whether WCH needs treatment [1]. WCH is a risk factor for developing sustained hypertension (SH), target organ damage (TOD), and may lead to cardiovascular (CV) events. WCH is also associated with metabolic syndrome, with increased risk of developing CV disease and diabetes [3,4]. Psychological characteristics like anxiety are responsible for the presence of WCH; however, more studies are required to understand this relationship. Guidelines developed by the ESH for WCH management suggest that those individuals with WCH without additional CV risk factors should be managed with lifestyle changes and monitored thereafter due to the increased risk of developing SH and TOD. Individuals with WCH with elevated CV risk or hypertension-mediated organ damage should be managed with lifestyle modifications along with drug therapy [1]. In this review, we discuss the etiology, prevalence, and treatment in primary care and also discuss the barriers to care and the importance of the primary care physician’s role in adequately managing individuals with WCH.

Definition

WCH, also known as isolated clinical hypertension, is a condition in which there is elevated BP in the office or clinic setting and normal out-of-office BP measured by ambulatory BP monitoring (ABPM) or home BP monitoring (HBPM) in individuals who have not received antihypertensive treatment. The American College of Cardiology (ACC) and American Heart Association (AHA) set guidelines in which WCH is defined as an office BP ≥ 130/80 mmHg but <160/100 mmHg and a daytime BP on ABPM or HBPM of <130/80 mmHg [5]. The ESH defines WCH as office BP of at least 140/90 mmHg and mean 24-hour BP of less than 130/80 mmHg in untreated individuals. This is different from the original definition, which took into consideration daytime ABPM and not the 24-hour BP monitoring. The updated definition includes nighttime BP, which is a better predictor of outcomes than daytime BP. The white coat effect is characterized by elevated BP in the clinic and lower home or ambulatory BP in both untreated and treated patients. The white coat effect is clinically significant if the difference between clinic and out-of-office BP is more than 20/10 mmHg [1]. There is no universal definition to diagnose WCH, and guidelines vary from ESH, ACC/AHA, and the National Institute for Health and Care Excellence (NICE) [1]. Variation also exists for the timeline to diagnose WCH after elevated office BP. European position paper suggests confirming diagnosis within a period of three to six months after office BP readings and conducting ABPM or HBPM annually to find progression for the development of SH. The Canadian diagnostic algorithm has recommendations for HBPM or ABMP after the first office visit to enable the detection of WCH early. HBPM has high specificity but low sensitivity for diagnosing WCH, indicating that HBPM is most useful for use over five years for the diagnosis of hypertension [5]. According to Kronish et. al., in 2015, the US Preventive Services Task Force (USPSTF) updated their hypertension screening recommendations to advise that patients with elevated office BP undergo ambulatory (ABPM) or HBPM prior to hypertension diagnosis. This recommendation is of importance as one in five patients with elevated office BP has normal out-of-office BP, which is known as white-coat hypertension. This can lead to unnecessary treatment with antihypertensive medications in individuals with WCH and is associated with avoidable health care costs [6]. There is variability in BP measurement based on the methods used to record the BP. Poor BP measuring techniques, such as not resting enough before the BP measurement, sitting crossed-legged, and a small cuff size, can lead to spurious elevation of BP, which could simulate WCH (falsely positive WCH) [1]. For individuals taking antihypertensive medications, the terms used are “treated white coat hypertension” or “white coat uncontrolled hypertension” [7]. WCH is associated with metabolic syndrome, with symptoms like obesity, high triglycerides, elevated BP, and glucose intolerance. A prospective study that followed men aged 50 years and older with WCH found that, as compared to normotensive (NT) men, they had higher BMI after a 20-year follow-up, higher triglycerides at initial appointment, and higher plasma glucose than NT men [2-4,8].

**Table 1:** Definition of WCH according to ESH, ACC/AHA, and NICE ACC, American College of Cardiology; AHA, American Heart Association; ESH, European Society of Hypertension; NICE, National Institute for Health and Care Excellence; WCH, white coat hypertension (Reference: PMCID: PMC7318827. 10.1177/1753944720931637)

Definition of WCH	
ESH	Clinic BP of 140/90 mmHg or higher and mean 24-hour BP of less than 130/80 mmHg
ACC/AHA	Clinic BP of 130/80 mmHg or higher and daytime ambulatory or home BP of less than 130/80 mmHg
NICE	Clinic BP of 140/90 mmHg or above and daytime ambulatory or home BP of less than 135/85 mmHg

Prevalence and risk factors

According to Cobos et. al., the prevalence of WCH has not been consistent across studies because of different BP cut-offs used for normal out-of-office BP readings and because of patients being inaccurately diagnosed with hypertension [2]. A meta-analysis of studies involving both treated and untreated patients estimates the prevalence of approximately 13%. This meta-analysis took the cutoff for diagnosis of WCH as 140/90 mmHg office BP and 135/85 mmHg for out-of-office BP, which are consistent with the NICE guidelines but different from the ESH and ACC/AHA guidelines. According to the Spanish Ambulatory BP Monitoring Registry, which followed the ESH guidelines, 35% untreated patients with elevated office BP were categorized as WCH. The prevalence in other studies is highly variable, ranging from 10% to 50% due to differences in the definition of WCH and the cohort studied [1]. However, there is low reproducibility of clinic BP measurements as only 55% of individuals meet the criteria of WCH when clinic and ambulatory BP recordings are taken at different time points [1]. Another review reported that 30% to 40% of patients diagnosed with hypertension solely on the basis of the office BP had normal out-of-office BP by ABPM. Hence, ABPM is important to distinguish patients with persistent hypertension from those with WCH [2,9,10]. ABPM should be used to confirm the diagnosis of WCH within three months and then repeated every six months to monitor for the progression to SH [2,11]. Some studies suggest that females, people over 50 years, and nonsmokers are more likely to develop WCH [2]. Women may experience a high level of stress and different stress responses compared to men in clinical visits. However, some studies suggest that a patient’s gender without the associated confounders of stress and anxiety does not determine if any one gender is more predisposed to WCH [2]. Older age is a predisposing factor for WCH due to an increase in arterial stiffness [2]. WCH is more prevalent in older age, accounting for ≥50% of hypertensive patients [12]. WCH is less likely to be found in smokers, as smoking is associated with nicotine exposure, which leads to an elevation of BP. That leads to elevation of both office BP as well as higher ambulatory BP ABPM compared to non-smokers, and hence less likelihood of having white coat effect [13].

Dysmetabolic risk factors

WCH is associated with an increase in body mass index, impaired fasting glucose, type 2 diabetes, metabolic syndrome, hypercholesterolemia, elevated uric acid levels, and lower HDL compared to NTs but lower than those with SH [12]. WCH is associated with subclinical alterations of organ structure and function compared to NT individuals, but less frequently than those with SH. This is supported by the findings of the Pressioni Arteriose Monitorate e Loro Associazioni (PAMELA) study that the prevalence of left ventricular hypertrophy (LVH) (by detecting body surface area-calculated left ventricular mass index >99 in women and >114 g/m2 in men) was minimal in NTs (4.2%), intermediate in WCH (20.4%) and maximum in SH (32.3%) and the odds ratio of having this condition in WCH after adjusting for age and sex, was 3.6 (95% CI, 2.2-5.9) and in SH is 4.3 (95% CI, 2.5-7.4) compared to NTs [12,14]. WCH is also more frequently associated with left ventricular diastolic dysfunction, shown by reduced E/A ratio (where E is the early ventricular relaxation and A is ventricular filling due to arterial contraction), left atrium enlargement, carotid intima-media thickening, plaques, increased urinary protein excretion, and silent cerebral infarction [12,15-19]

## Review

Methods

For this research, we searched through the last 25 years of articles from PubMed, Web of Science, Google Scholar, and Scopus and selected articles mentioning WCH. Keywords/search terms included were “white coat hypertension”, “white coat effect”, “ambulatory blood pressure monitoring”, and “out-of-office blood pressure”. One researcher collected the initial collection of material, another researcher reviewed the content, and the third researcher went through the manuscript and made sure that the research mentioned in the manuscript was referenced appropriately. We have outlined the importance of monitoring BP in patients with elevated BP in the clinic setting and measures taken by the PCPs to manage WCH.

Treatment in primary care

There is insufficient evidence from randomized controlled trials on whether WCH needs treatment [1]. The ESH has outlined the guidelines for WCH management. According to the ESH, patients with WCH and no additional CV risk factors are advised to follow lifestyle modifications along with close monitoring, as they have an increased risk of developing SH and TOD. For patients with WCH and hypertension-mediated organ damage or increased CV risk, management should include lifestyle interventions along with pharmacotherapy [1]. The latest US guidelines do not recommend any treatment for WCH due to the presence of normal out-of-office BP readings. Hence, after the patient is diagnosed with WCH, lifestyle changes should be implemented and recommended for annual ABPM or HBPM to detect progression to SH [20]. According to the PAMELA population study, individuals with WCH had a 2.5-fold higher risk of developing SH after 10 years compared with NT individuals. The systolic BP (SBP) at baseline was the main predictor of progressing to SH, and the diastolic BP (DBP) was attenuated, thus resulting in an increase in pulse pressure. Hence, progression to SH can be attributed to stiffening of larger arteries [1,21]. TOD can be assessed by measuring the carotid intima-media thickness (CIMT) or LVH. Patients with WCH have significantly higher mean CIMT than those who are NT and significantly lower than those with SH (p < 0.05) after adjusting for age and other risk factors, including smoking, diabetes mellitus, and hyperlipidemia [1,22]. Studies have found an increase in left ventricular mass in those with WCH compared with NT patients [15]. However, there are very few large, prospective, randomized control trials evaluating the effect of anti-hypertensive treatment on the incidence of CV and cerebrovascular events in patients with WCH [1]. A study from Cohen et al. showed that untreated WCH was associated with increased risk of CV events and mortality, compared to NT individuals [23]. However, another study by Pierdoemnico et al. reported no difference in CV risk between WCH and NT [1,24]. In one study, ABPM was performed on 497 outpatients who were being treated for hypertension. It was found that 63% of these were resistant hypertensive patients, and they were unsuccessfully treated, and 37% had normal ambulatory BP measurement and were having WCH. Hence, it is important not to start treatment for patients with mild to moderate high BP unless there is TOD and the BP remains high after three to six visits [2,25-27]. In one prospective double-blinded antihypertensive treatment trial, the European Lacidipine Study on Atherosclerosis (ELSA) trial, around 2200 patients with moderate elevations of both systolic and diastolic BP were followed over four years. They had office and ambulatory BP (ABP) measured and then were randomized to treatment and followed at six-month office BP and 12-month ABP intervals during treatment. The ELSA trial compared the effects of long-term β-blocker or calcium channel blocker (with additional diuretic if required) treatment on progression of carotid-intima-media thickness in mild to moderate essential hypertension. Patients were randomized to either a morning dose of atenolol 50 mg or lacidipine 4 mg. The morning dose was increased to 100 mg atenolol and 6 mg lacidipine, if after one month, office diastolic BP was not lower than 95 mmHg. If the office BP still remained uncontrolled after two months, hydrochlorthiazide was administered at two progressive doses of 12.5 mg and 25 mg QD. Antihypertensive treatment effectively reduced office BP in WCH; however, the treatment did not lower the 24-hour mean systolic and diastolic BP in WCH and even showed a small increase from the first to the last year of antihypertensive treatment. However, the 24-hour BP was effectively reduced in patients with SH [28]. Hence, it is important for further randomized outcome-based trials to establish whether BP-lowering medications have any benefit in WCH, as there is no BP reduction in the 24-hour BP [28].

Barriers to Out-of-Office Blood Pressure Monitoring and Scope for Improvement in Primary Care

There are several barriers to the implementation of ambulatory and HBPM. In one survey, primary care providers from two geographic regions in the United States (Southern states, mainly Alabama, and New York City) were asked about the barriers they faced in practice in using ABPM to exclude WCH. Barriers were categorized according to the Theoretical Domains Framework (TDF), which assessed barriers affecting behavioral changes across varied domains such as knowledge, cognitive skills, professional role, environmental resources, social factors, behavioral factors, motivation, and goals. The most important barrier to ordering ABPM (48.2%) was environmental resources and context, which included challenges such as out-of-pocket costs, insurance coverage for the test, ABPM equipment cost, time taken by the staff for conducting the test, inadequate time for physicians to implement the ABPM protocol, and lack of access to ABPM. The second highest domain (37.9%) was related to the provider’s beliefs regarding their own capability of obtaining ABPM data due to patients incorrectly following protocol by monitoring on the incorrect day or using a non-validated ABPM device. The cognitive skills domain comprised 10.7% of votes, with provider concerns regarding skills training to perform ABPM and interpreting the results for patients. The knowledge domain comprised 3.3% of responses with provider concerns regarding inadequate knowledge of recommendations for out-of-office BP testing. The most important barriers to HBPM were concerns about patient non-compliance, leading to validity concerns of HBPM test results, along with concerns regarding provider skills and knowledge [6]. Evidence-based behavior changes and policy recommendations can increase the implementation of recommendations. Evidence-based behavior changes include education, persuasion, incentivization, enablement, modeling, and environmental restructuring. Policies that improve incentives for out-of-office testing for ABPM should be implemented, as it is currently not accessible to many primary care providers in the USA. Allied health professionals can be trained to teach patients to perform HBPM to address the concern of insufficient provider time [6].

Strategies for Improved White Coat Hypertension Management

WCH is likely associated with the patient’s anxiety in the clinic in the presence of the physician. Research suggests that improving the patient-physician relationship can decrease patient anxiety, resulting in a lower likelihood of WCH [2]. Research suggests that there is situational anxiety involved, rather than persistent anxiety, when the patient has expectations of elevated BP while being measured, resulting in elevated BP. Patients may adopt the “sick role” wherein they find consistency in their symptoms and diagnosis, thus demonstrating high BP due to anxiety when visiting the doctor, or they may anticipate more negative information, leading to anxiety and demonstrating WCH [2,29]. Primary care physicians can play a vital role by not labeling or diagnosing a patient as hypertensive based only on office readings, as this can increase the likelihood of WCH due to experiencing a greater anxiety level during future office visits [2]. Effective communication and building a trusting relationship with the patient can reduce the patient’s anxiety. It is important to ask open-ended questions, demonstrate empathy, and discuss patients’ psychosocial issues, also referred to as “bedside manner” or “relational communication” [2,30,31]. Primary care physicians can also take training in communication skills to improve empathy towards patients. A study demonstrated that training in communication skills resulted in a 37% increase in rating of physician empathy from baseline to follow-up [2,32]. Time constraints can affect the physician-patient relationship and make patients feel more anxious. It is important to build a trusting relationship with the patients by providing adequate time with patients, understanding their perspective regarding illness, and answering their questions [2,33]. Training can also be provided to patients, which helps in patient participation and involvement and helps them prepare before the physician visit by listing questions, thus reducing their anxiety [2,34,35]. Telemedicine, with the help of email communication and interactive video medical visits, can help reduce anxiety and WCH. Telemedicine service using an automatic home BP monitor linked to a telephone can enable patients to send their BP readings directly to their physician. Results of one study showed that 64% of patients using this service were diagnosed with essential hypertension, whereas only 26% patients not using this service were accurately diagnosed with hypertension. It also helped diagnose hypertension earlier. Although telemedicine has not been used with WCH, it can benefit patients with WCH [2,36,37]. Use of in-office automated BP devices, which can take multiple BP measurements without the presence of a physician or health care provider, can help to reduce the prevalence of WCH as well as lessen the need for ABPM [13].

## Conclusions

In this research article, we covered WCH, the white coat effect, its prevalence, and treatment requirements. WCH is a common medical condition in which patients have elevated BP in the clinic setting and normal BP in an out-of-office environment. Due to this condition, it can be misdiagnosed and unnecessarily treated as persistent hypertension. WCH is an under-researched phenotype of SH, and it is not clear whether it is a benign condition. There is a need for more long-term prognostic studies; however, the challenge lies in poor reproducibility over time. WCH is associated with patients' anxiety, and we have covered the importance of the primary care physician’s role in managing WCH by adopting effective communication skills to reduce the associated anxiety of the patient and not labeling the patient as hypertensive on the basis of BP measurement on the initial visit. Limitations of ambulatory BP and HBPM are discussed. The article also discusses the scope of improvement with the help of policy recommendations and changes, which can help improve out-of-office testing for ABPM, along with behavioral changes and telemedicine. Policy changes that increase incentives for ABPM are needed, as it is not currently accessible to many primary healthcare providers in the United States. Barriers regarding primary providers' beliefs about their own capabilities can be addressed using a case-based curriculum with grand rounds and continuing medical education presentations. The article has also discussed the antihypertensive medications in the management of WCH. There is a need for further randomized outcome-based trials to evaluate the effects of anti-hypertensive treatment on the incidence of CV and cerebrovascular outcomes and study whether there are any benefits of anti-hypertensive medications in WCH.
